# Divergence of gut permeability and mucosal immune gene expression in two gluten-associated conditions: celiac disease and gluten sensitivity

**DOI:** 10.1186/1741-7015-9-23

**Published:** 2011-03-09

**Authors:** Anna Sapone, Karen M Lammers, Vincenzo Casolaro, Marcella Cammarota, Maria Teresa Giuliano, Mario De Rosa, Rosita Stefanile, Giuseppe Mazzarella, Carlo Tolone, Maria Itria Russo, Pasquale Esposito, Franca Ferraraccio, Maria Cartenì, Gabriele Riegler, Laura de Magistris, Alessio Fasano

**Affiliations:** 1 Department of Internal and Experimental Medicine Magrassi-Lanzara, Seconda Università degli Studi di Napoli, Naples, Italy; 2 Mucosal Biology Research Center, University of Maryland School of Medicine, Baltimore, MD, USA; 3 Johns Hopkins Asthma and Allergy Center, Johns Hopkins University School of Medicine, Baltimore, MD, USA; 4 Department of Experimental Medicine, Seconda Università di Napoli, Naples, Italy; 5 Institute of Food, Consiglio Nazionale delle Ricerche (CNR), Avellino, Italy; 6 Department of Pediatrics, Seconda Università degli Studi di Napoli, Naples, Italy; 7 Servizio di Endoscopia Digestiva, Seconda Università degli Studi di Napoli, Naples, Italy; 8 Morfopatologia, Seconda Università degli Studi di Napoli, Naples, Italy

## Abstract

**Background:**

Celiac disease (CD) is an autoimmune enteropathy triggered by the ingestion of gluten. Gluten-sensitive individuals (GS) cannot tolerate gluten and may develop gastrointestinal symptoms similar to those in CD, but the overall clinical picture is generally less severe and is not accompanied by the concurrence of tissue transglutaminase autoantibodies or autoimmune comorbidities. By studying and comparing mucosal expression of genes associated with intestinal barrier function, as well as innate and adaptive immunity in CD compared with GS, we sought to better understand the similarities and differences between these two gluten-associated disorders.

**Methods:**

CD, GS and healthy, gluten-tolerant individuals were enrolled in this study. Intestinal permeability was evaluated using a lactulose and mannitol probe, and mucosal biopsy specimens were collected to study the expression of genes involved in barrier function and immunity.

**Results:**

Unlike CD, GS is not associated with increased intestinal permeability. In fact, this was significantly reduced in GS compared with controls (*P *= 0.0308), paralleled by significantly increased expression of claudin (CLDN) 4 (*P *= 0.0286). Relative to controls, adaptive immunity markers interleukin (IL)-6 (*P *= 0.0124) and IL-21 (*P *= 0.0572) were expressed at higher levels in CD but not in GS, while expression of the innate immunity marker Toll-like receptor (TLR) 2 was increased in GS but not in CD (*P *= 0.0295). Finally, expression of the T-regulatory cell marker FOXP3 was significantly reduced in GS relative to controls (*P *= 0.0325) and CD patients (*P *= 0.0293).

**Conclusions:**

This study shows that the two gluten-associated disorders, CD and GS, are different clinical entities, and it contributes to the characterization of GS as a condition associated with prevalent gluten-induced activation of innate, rather than adaptive, immune responses in the absence of detectable changes in mucosal barrier function.

## Background

Gluten is the structural protein component of the grains wheat, rye and barley, which are the basis for a variety of flour- and wheat-derived food products consumed throughout the world. Possibly, the introduction of gluten-containing grains, which occurred about 10,000 years ago with the advent of agriculture, represented a "mistake of evolution" that created the conditions for human diseases related to gluten exposure, the best known of which are mediated by the adaptive immune system: wheat allergy and celiac disease (CD). In both conditions, the reaction to gluten is mediated by T-cell activation in the gastrointestinal mucosa. However, in wheat allergy, it is the cross-linking of immunoglobulin E (IgE) by repeat sequences in gluten peptides (for example, Ser-Gln-Gln-Gln-(Gln-)Pro-Pro-Phe) that triggers the release of chemical mediators, such as histamine, from basophils and mast cells [1]. In contrast, CD, which affects approximately 1% of the general population, is an autoimmune disorder, as heralded by the appreciation of specific serologic markers, most notably serum antitissue transglutaminase (tTG) autoantibodies, by the autoimmune enteropathy that characterizes this condition and by autoimmune comorbidities.

Besides CD and wheat allergy, there are cases of gluten reactions in which neither allergic nor autoimmune mechanisms are involved. These are generally defined as gluten sensitivity (GS) [2-5]. Some individuals who experience distress when eating gluten-containing products and show improvement when following a gluten-free diet may have GS instead of CD. GS patients are unable to tolerate gluten and develop an adverse reaction when eating gluten that usually, and differently from CD, does not lead to small intestinal damage. While the gastrointestinal symptoms in GS may resemble those associated with CD, the overall clinical picture is generally less severe and is not accompanied by the concurrence of tTG autoantibodies or autoimmune disease. Typically, the diagnosis is made by exclusion, and an elimination diet and "open challenge" (that is, the monitored reintroduction of gluten-containing foods) are most often used to evaluate whether the patient's health improves with the elimination or reduction of gluten from the diet.

A number of *in vitro *studies have confirmed the cytotoxicity of gluten's main antigen, gliadin. Gliadin has agglutinating activity, reduces F-actin content, inhibits cell growth, induces apoptosis, alters redox equilibrium and causes a rearrangement of the cytoskeleton through the zonulin pathway and the loss of tight junction (TJ) competence in the gastrointestinal mucosa [6-9]. The diversity of gluten-induced conditions is in line with the notion that the immune system reacts to and deals with the triggering environmental factor, gliadin, in distinct ways. In the present study, we sought to gain initial knowledge on intestinal barrier function and the immune response to gluten in patients with GS. Specifically, we were interested in understanding to what extent innate and adaptive immune pathways are activated in GS compared to CD. To achieve these aims, we looked at the mucosal expression of genes associated with intestinal barrier function and immune parameters known or implied to be aberrantly regulated in CD. The results provide the first documentation to date of genes and pathways possibly involved in the pathogenesis of GS, and, at the same time, contribute to improving our understanding of the processes leading to CD and other autoimmune phenomena.

## Methods

### Definition of GS

GS patients are defined as those patients in which CD, wheat allergy and other clinically overlapping diseases (type 1 diabetes, inflammatory bowel diseases and *Helicobacter pylori *infection) have been ruled out and whose symptoms were triggered by gluten exposure and alleviated by gluten withdrawal. All enrolled patients underwent a gluten challenge carried out for approximately 4 months under clinical supervision. At the end of the challenge, patients underwent CD serology screening, Human Leukocyte Antigen (HLA) *DQ2/DQ8 *typing and an upper endoscopy with duodenal biopsies. Once endoscopies were performed, patients were placed back on a gluten-free diet and their symptoms monitored over time. GS were considered those patients with negative autoantibody serology (endomysium antibodies-immunoglobulin A (EMA-IgA) and tTG-IgA), normal mucosa (Marsh stage 0) or increased intraepithelial lymphocytes (Marsh stage 1) and improvement of symptoms within days of the implementation of the diet. To avoid any possible selection bias and to prove that these patients are different from CD patients, we elected to enroll every patient fulfilling the above-described definition of gluten sensitivity.

### Subjects

Consent was obtained from all enrolled subjects after the nature of the investigation was explained and in accordance with the approved protocol from the Institutional Review Board at the University of Naples. A total of 26 GS patients diagnosed according to the criteria outlined above were enrolled. For comparison, 42 patients with active CD were recruited according to the modified 2004 criteria of the European Society of Pediatric Gastroenterology, Hepatology and Nutrition (ESPGHAN) [10]. Finally, 39 control subjects were enrolled from among individuals undergoing upper endoscopy for dyspepsia. We limited our control group to individuals who had exclusively dyspeptic symptoms and no underlying inflammation as proven by normal erythrocyte sedimentation rate, C-reactive protein and mucoprotein evaluation. These subjects did not have CD or GS and are referred to hereinafter as dyspeptic controls (DC).

Subject characteristics are summarized in Table 1. For each set of experiments, a representative subgroup of CD, GS and DC were analyzed on the basis of the subjects' consent to participate in part or all of the proposed studies and on material transfer agreements between the institutions involved in this project. At least six duodenal biopsies were collected for histological evaluation and gene expression. No selection bias based on sex, age or type of symptoms was observed.

**Table 1:** Clinical and laboratory characteristics of the study subjects^a^

Characteristics	DC	CD	GS
Number of samples	39	42	26
Age at diagnosis, yr (mean ± SD)	33.36 ± 10.94	26.88 ± 11.77	30.73 ± 12.19
Sex (F/M)	23/16	32/10	17/9
Intestinal symptoms	Dyspepsia	Chronic diarrhea Abdominal pain Weight fluctuation Weakness Smelly, fatty stools	Diarrhea Abdominal pain Weight loss Gas
Extraintestinal symptoms	None	Bone or joint pain Osteoporosis Behavioral changes Tingling leg numbness Muscle cramps Missed menstruation Infertility Recurrent miscarriage Delayed growth Thyroiditis Tooth discoloration Unexplained anemia	Bone or joint pain Osteoporosis Leg numbness Muscle cramps Unexplained anemia Glossitis
EMA-IgA	All negative	34 positive 3 negative 5 not determined	All negative
tTG-IgA	All negative	37 positive 5 negative	All negative
AGA-IgA/IgG	All negative	29 positive 10 negative 3 not determined	12 positive 13 negative 1 not determined
MHC profile	None determined	22 DQ2 and/or DQ8 positive 2 DQ2 and/or DQ8 negative 18 not determined	12 DQ2 and/or DQ8 positive 9 DQ2 and/or DQ8 negative 5 not determined
Wheat IgE	1 positive 37 negative 1 not determined	18 negative 24 not determined	22 negative 4 not determined

^a^DC, dyspeptic controls; CD, celiac disease; GS, gluten-sensitive individuals; SD, standard deviation; EMA-IgA, endomysium antibodies-immunoglobulin A; tTG-IgA, tissue transglutaminase-immunoglobulin A; AGA-IgA/IgG, anti-gliadin antibodies-immunoglobulin A/immunoglobulin G; MHC, major histocompatibility complex; IgE, immunoglobulin E.

### Determination of intestinal permeability

*In vivo *permeability was determined by means of the lactulose/mannitol (LA/MA) test as previously described [11]. The detection and measurement of the two sugar probes in the urine was performed by high-performance anion exchange chromatography coupled with pulsed amperometric detection, which permits direct quantification of nonderivative carbohydrates on a Dionex model DX-500 with a gradient pump module GP40 and sample loop of 50 μl. Samples were loaded onto CarboPac PA-100 guard columns and eluted with a NaOH-NaAc gradient (Dionex, Sunnyvale, CA, USA).

### Histology and immunohistochemistry of jejunal biopsies

Serial sections (4 μm) were prepared from duodenal formalin-fixed, paraffin-embedded biopsies. Biopsy specimens were staged by histology according to the Marsh classification [12,13]. Immunostaining to identify intraepithelial lymphocytes (IELs) was performed as described elsewhere [14]. Acetone-fixed sections (5 μm) were stained with monoclonal antibodies for CD3 (1:200; Dako, Milan, Italy) and T-cell receptor γδ (TCRγδ) (1:50; Thermo Scientific, Rockford, IL, USA) using the peroxidase-antiperoxidase protocol. The sections were finally stained with Mayer's hematoxylin (Sigma-Aldrich, St. Louis, MO, USA). IEL density was calculated as the percentage of enterocytes (ECs). All slides were analyzed by two observers who were blinded to these procedures.

### Determination of mucosal gene expression

Intestinal gene expression was measured by quantitative real-time polymerase chain reaction (qPCR) assay as described previously [15]. Small intestinal biopsies from participating subjects were homogenized and total RNA was extracted using TRIzol reagent (Invitrogen, Grand Island, NY, USA). Primers and probes for qPCR of specific transcripts and of the *18S *gene as a housekeeping control were purchased from Applied Biosystems (Foster City, CA, USA). qPCR was performed using the TaqMan protocol and the Applied Biosystems 7500 Fast Real-Time PCR System. Amplification conditions were as follows: 50°C for 2 minutes and 95°C for 10 minutes, followed by 50 cycles at 95°C for 15 seconds and 60°C for 1 minute. The data were calculated as the change in cycle threshold (Δ_CT_) of the gene of interest to *18S *and are expressed as percentages of *18S *(that is, 2^-ΔCT ^× 100).

### Statistical analysis

All data were analyzed and graphed using Prism version 5.0 software (GraphPad Software, La Jolla, CA, USA). Most of the variables examined in this study appeared to be arranged in a non-normal, positively skewed distribution, making conventional parametric statistics misleading. Therefore, data are described by the medians and interquartile ranges (IQRs), and the degree of variability among groups was determined using the Kruskal-Wallis nonparametric algorithm. A two-tailed Mann-Whitney *U *test (MWU) was used for pairwise comparisons. The level of significance was set at *P *< 0.05.

## Results

### Clinical and laboratory characteristics of GS subjects

The patients who fulfilled the GS diagnostic criteria (see Methods section) experienced symptoms overlapping those presented by CD patients (see Table 1), but their symptoms resolved within a few days after the implementation of the gluten-free diet, and they remained symptom-free for the entire follow-up period (up to 4 years). Interestingly, 48% of GS patients were anti-gliadin antibody (AGA)-positive, and 57% were *HLA-DQ2*-positive and/or *HLA-DQ8*-positive. Fifty-six percent of AGA-positive GS patients were *HLA-DQ2*-positive and/or *HLA-DQ8*-positive, while the remaining 44% were *HLA*-negative, suggesting that AGA production was not associated with a *HLA-DQ2*-restricted and/or *HLA-DQ8*-restricted presentation.

### Intestinal permeability

**Figure 1:**
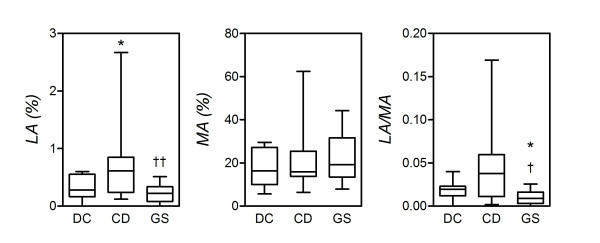
**Reduced intestinal permeability in gluten-sensitive (GS) patients**. Small intestinal permeability was probed by measuring the urinary cumulative 5-hour amount of lactulose (LA) (percentage of ingested), mannitol (MA) and the LA-to-MA ratio as described in (see Methods). Boxes represent the medians and interquartile ranges, and whiskers represent the range of independent determinations in 13 GS patients, 11 celiac disease (CD) patients, and 14 dyspeptic controls (DC) (see intestinal permeability section of the text, pages 8-9). *P < 0.05 relative to DC and ^†^*P *< 0.05 relative to CD (Mann-Whitney *U *test).

While CD is consistently associated with impaired mucosal barrier function and increased small intestinal permeability, it is not known whether patients with GS present similar alterations. To address this question, the LA/MA urinary ratio was determined in the three study groups. The LA/MA ratio, and hence small intestinal permeability, varied significantly among the three groups (*P *= 0.0113; Kruskal-Wallis test). In particular, it was significantly higher in CD patients compared to GS patients (*P *= 0.0138; MWU). A similar difference between CD patients and DC only approximated significance (*P *= 0.0950). Interestingly, the LA/MA ratio in GS patients was also significantly reduced relative to DC (*P *= 0.0308) (Figure 1).

Figure 1 shows that the increase in intestinal permeability in CD patients, relative to DC, was ascribed to a significantly increased urinary concentration of lactulose (*P *= 0.0400), with no significant changes in mannitol titers (*P *= 0.4937; not significant). Since the urinary recovery of lactulose reflects its exclusive transport through the paracellular pathway, while mannitol is a marker of the transcellular pathway [11], this confirms that the changes in LA/MA ratio observed in CD reflect increased paracellular permeability. Likewise, the reduced LA/MA ratio in GS patients relative to CD and DC appeared to be exclusively determined by reduced lactulose titers, a finding again in line with the involvement of processes regulating macromolecular passage through the paracellular pathway. Specifically, the median percentage of urinary lactulose in GS was significantly lower than that in CD (*P *= 0.0049), while the difference between GS and DC was not significant (*P *= 0.1817; not significant).

### Small intestinal expression of TJ proteins

**Figure 2:**
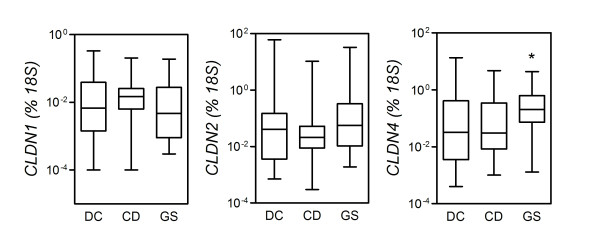
**The relative expression of tight junction (TJ)-related genes in the mucosa of gluten-sensitive patients (GS) versus celiac disease patients (CD)**. Expression of the indicated genes encoding for components of the TJ complex was measured by quantitative polymerase chain reaction assay of RNA extracted from small intestinal bioptic specimens (see Methods). Boxes represent the median (interquartile ratio), and whiskers represent the range of relative RNA levels, expressed as a percentage of an *18S *housekeeping gene in 24 GS patients, 31 CD patients and 24 dyspeptic controls. *P < 0.05 relative to CD (Mann-Whitney *U *test).

To understand whether the changes in intestinal permeability observed in CD and GS are associated with altered expression of genes encoding for key TJ components, the expression of claudins (CLDN), TJ protein (TJP)-1 (also known as zonula occludens-1) and occludin (OCLN) was determined in intestinal tissues obtained by upper gastrointestinal endoscopy. Biopsy specimens were immediately processed for mRNA extraction, and the transcript levels of the genes *CLDN1*, *CLDN2*, *CLDN3*, *CLDN4*, *TJP1 *and *OCLN *were measured by qPCR. As shown in Figure 2, *CLDN4 *was expressed at significantly higher levels in GS than in CD (*P *= 0.0286), while a similar difference with DC almost reached significance (*P *= 0.0565). Similar levels of *CLDN4 *were detected in biopsies from DC and CD (*P *= 0.7279; not significant). Likewise, no significant differences were observed across the three groups in the mRNA levels of *CLDN1 *or *CLDN2 *or the other TJ-related genes examined (not shown). Although this might be due to the limited number of cases studied, it is unlikely that inclusion of a larger sample would compensate for the high level of variability and skewness encountered within each group.

### Intraepithelial lymphocytes

**Figure 3:**
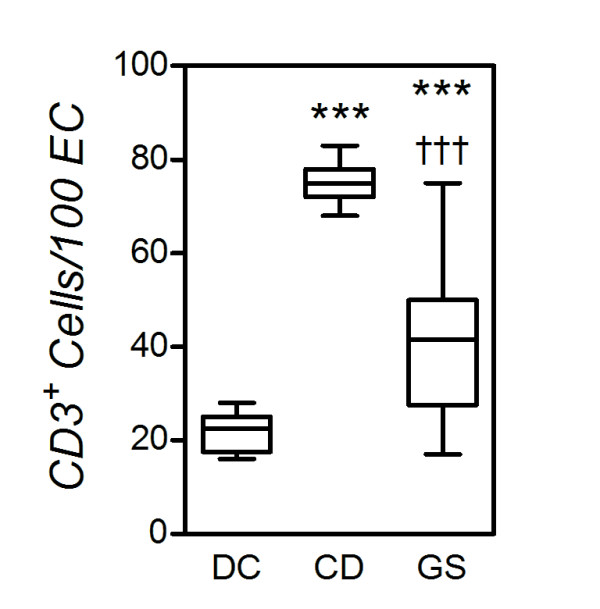
**Reduced numbers of intraepithelial lymphocytes (IELs) in gluten-sensitive patients (GS) versus celiac disease patients (CD)**. Small intestinal bioptic specimens were stained for the T-cell marker CD3 in immunohistochemistry, and CD3^+ ^cells are enumerated as indicated in Methods. Boxes represent the median (interquartile range), and whiskers represent the range of IEL numbers relative to 100 enterocytes in the same samples in 16 GS, 11 CD and 12 dyspeptic controls (DC). ***P < 0.0005 relative to DC; ^†††^*P *< 0.0005 relative to CD (Mann-Whitney *U *test).

The GS patients recruited in this study did not present relevant autoimmune phenomena and autoimmune serology (Table 1). In addition, in this group of patients, we did not detect a clear-cut association with the major histocompatibility complex (MHC) haplotype, as extensively documented in CD patients. These clinical findings led us to hypothesize that the adaptive immune system may not be as critically involved in GS as it is in CD (or in wheat allergy). To substantiate this argument, our end points included markers of adaptive and innate immune responses in the small intestinal mucosa of these subjects. In a first series of observations, we compared the numbers of IELs in GS versus CD patients. As recently reported by our group [15], histology revealed a normal to mildly inflamed mucosa (Marsh stage 0 or 1) in GS patients, while all CD patients showed partial or subtotal villous atrophy with crypt hyperplasia according to the ESPGHAN criteria [10]. The results of CD3 immunohistochemistry are summarized in Figure 3. The numbers of CD3^+ ^IELs varied considerably among the three groups (*P *< 0.0001; Kruskal-Wallis test). As anticipated, CD patients had increased numbers of CD3^+ ^IEL relative to DC (*P *< 0.0001; MWU). CD3^+ ^IELs were significantly more numerous in GS patients than in DC (*P *< 0.0001), but significantly less so than in CD (*P *= 0.0012). Notably, though, all but four GS patients had IEL numbers above the currently accepted range of normality (≤30/100 EC), suggesting an intermediate-level, yet pathogenetically significant, involvement of the adaptive immune system in this condition. On the other hand, the number of TCRγδ IELs was consistently elevated in CD (>3.4/100 ECs), while the numbers in GS and DC were similar (data not shown).

### Mucosal expression of adaptive immunity-related cytokines

A number of studies have documented the gluten-dependent rise in the systemic and mucosal expression of cytokines associated with Th1 and Th17 adaptive responses in CD [16-19]. We have recently shown that the Th17 signature cytokine, IL-17A, is expressed at significantly higher levels in the small intestinal mucosa of CD patients but not GS patients [15]. We have also shown that gliadin induces circulating monocytes to produce the Th17-active cytokine, IL-23, an effect mediated by concomitant expression of IL-1β and IL-6 [17,20]. To extend these observations and to further define the role of adaptive immunity in GS, mRNA extracted from small intestine biopsies was subjected to qPCR for expression of the pleiotropic, Th17-activating cytokine interleukin (IL)-6, the Th1 cytokine interferon (IFN)-γ, and IL-21, a Th1 cytokine involved both in the differentiation of Th17 cells and in sustaining ongoing Th1 responses [21].

**Figure 4:**
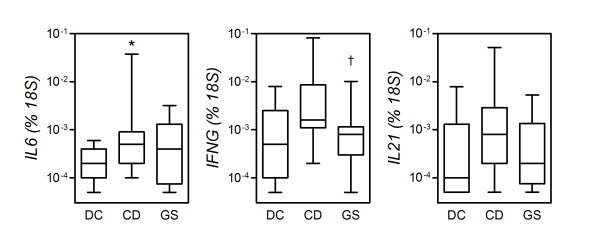
**Increased levels of transcripts for effector cytokines in the mucosa from celiac disease patients (CD) but not gluten-sensitive patients (GS)**. Expression of the indicated genes was measured by quantitative polymerase chain reaction assay (see Methods). Boxes represent the median (interquartile range) and whiskers represent the range of RNA levels relative to an *18S *housekeeping gene in 13 GS, 19 CD and 11 dyspeptic controls (DC). *P < 0.05 relative to DC (Mann-Whitney *U *test).

As shown in Figure 4, CD patients as a group expressed higher levels of transcripts for IL-6 (IL6), IFN-γ (*IFNG*) and IL-21 (IL21) relative to DC. However, because of a high degree of variability among CD donors and because of a subgroup effect already documented in our earlier report on IL-17A [15], only the difference in IL-6 expression was statistically significant (*P *= 0.0124), while the differences in *IFNG *and IL21 transcript levels approximated significance (*P *= 0.0700 and *P *= 0.0572, respectively). Levels of IL6, *IFNG*, and IL21 transcripts in specimens from GS patients did not differ significantly from those in DC or CD, except for a significant reduction in *IFNG *levels relative to CD (*P *= 0.0222). *IFNG *was the only cytokine gene tested that showed a significant degree of variability across the three groups (*P *< 0.0428; Kruskal-Wallis test). Thus, in line with its role in Th17 cell differentiation, IL-6 is expressed at significantly increased levels in CD mucosa, but not in GS mucosa. A concomitantly reduced expression of IFN-γ in GS relative to CD supports the notion of a lower-level involvement of the adaptive immune system in this condition.

### Small intestinal expression of Toll-like receptors

**Figure 5:**
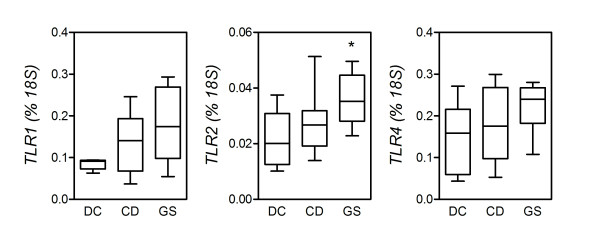
**Increased levels of transcripts for pattern recognition receptors in the mucosa from gluten-sensitive patients (GS) but not celiac disease patients (CD)**. Expression of the indicated genes encoding for Toll-like receptors TLR1, TLR2 and TLR4 was measured by quantitative polymerase chain reaction assay (see Methods). Boxes represent the median (interquartile range) and whiskers represent the range of RNA levels relative to an *18S *housekeeping gene in 8 GS, 12 CD and 5 dyspeptic controls (DC). *P < 0.05 relative to DC (Mann-Whitney *U *test).

It has been reported that the expression of Toll-like receptor 1 (*TLR1*), *TLR2*, and *TLR4* is increased in the small intestine mucosa of CD patients, lending support to the idea that innate immune phenomena may precede and/or accompany the progression of CD and other autoimmune conditions [22-24]. To preliminarily assess the involvement of innate immunity in GS, we compared the expression of these molecules in fresh biopsies from the three study groups. As illustrated in Figure 5, expression of *TLR1 *and *TLR2*, but not *TLR4*, mRNA was higher in CD patients than in DC, but neither of these differences reached significance. In contrast, relative to DC, patients with GS presented significantly higher levels of *TLR2 *transcripts (*P *= 0.0295), while transcript levels of *TLR1 *and *TLR4 *in GS were generally higher without reaching significance (*P *= 0.0932 and *P *= 0.1274, respectively).

### Mucosal expression of regulatory T-cell-associated genes

**Figure 6:**
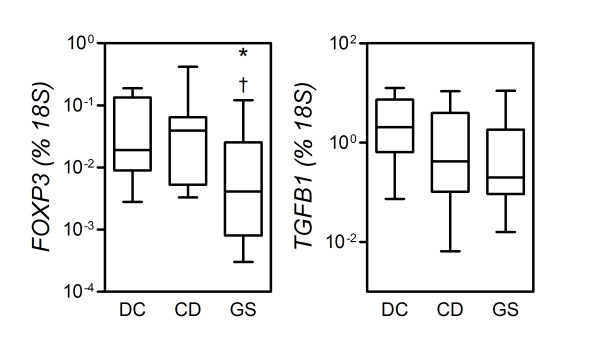
**Reduced levels of transcripts of immune regulatory genes in the mucosa from gluten-sensitive patients (GS), but not celiac disease patients (CD)**. Expression of the indicated genes was measured by quantitative polymerase chain reaction assay (see Methods). Boxes represent the median (interquartile range) and whiskers represent the range of RNA levels relative to an *18S *housekeeping gene in 19 GS, 12 CD and 8 dyspeptic controls (DC). *P < 0.05 relative to DC (Mann-Whitney *U *test).

To preliminarily assess whether a difference in immune regulatory function may account for the differential involvement of the adaptive system in CD versus GS patients, we measured mRNA expression of two regulatory T-cell (Treg) signature molecules, forkhead box P3 (FOXP3) and transforming growth factor-β_1 _(TGFB1), in small intestinal biopsy specimens. In contrast to earlier reports [25,26], the levels of expression of both FOXP3 and TGFB1 in CD patients did not differ significantly from those in DC (*P *= 0.8868 and *P *= 0.1535, respectively). As for TGFB1, there appeared to be a trend toward lower, rather than higher, levels of expression in CD patients (Figure 6). This phenomenon was more accentuated in GS patients, in whom expression of FOXP3 and TGFB1 was, significantly for FOXP3, reduced relative to DC (*P *= 0.0325 and *P *= 0.0710, respectively). FOXP3 reduction in GS accounted for a significant variation across groups (*P *< 0.0250; Kruskal-Wallis test) and was also significant relative to CD (*P *= 0.0293), while no significant difference was observed in TGFB1 expression in GS versus CD patients (*P *= 0.7832). These findings, quite opposite to our starting hypothesis but perhaps in line with earlier reports on CD and other conditions [27,28], may suggest a reduced level of activation and/or recruitment of Treg cells in the GS small intestine mucosa relative to gluten-tolerant controls.

## Discussion

CD, an autoimmune enteropathy, results from an inappropriate T-cell-mediated adaptive immune response against ingested gliadin. In the past few years, though, it has become apparent that "classic" CD represents the tip of the iceberg of an overall disease burden [4,29]. An emerging problem is the clinical characterization of a group of gluten-reactive patients, accounting for roughly 10% of the general population, presenting with symptoms similar to CD but with negative CD serology and histopathology. As in CD, these patients, here and elsewhere referred to as GS [15], experience distress when eating gluten-containing products and show improvement when following a gluten-free diet. Differently from CD, though, in GS the adverse reactions that develop while eating gluten are not followed by the appearance of autoantibodies and by persisting damage to the small intestine. Symptoms in GS may resemble some of the gastrointestinal symptoms that are associated with CD or wheat allergy, but objective diagnostic tests for this condition are currently missing. Therefore, a diagnosis of GS is commonly made by exclusion.

In itself, the absence of autoantibodies and intestinal lesions does not rule out the intrinsic toxicity of gluten, whose intake, even in non-CD individuals, has been associated with damage to other tissues, organs and systems besides the intestine [6,30,31]. In the present study, we have sought to identify functional, morphologic and immunologic parameters to help differentiate GS from CD and to preliminarily understand its pathophysiology. We report here for the first time evidence of differential intestinal mucosal responses to gluten in these two conditions.

We have shown that a normal to mild histology in GS is paralleled by a conserved barrier function. Indeed, small intestinal permeability, when tested with a LA/MA double sugar probe, was significantly lower in GS than in CD patients or even DC. Increased intestinal permeability is thought to be an early biological change that precedes the onset of several autoimmune diseases [32-34]. Loss of intestinal barrier function brings with it a continuous aberrant passage of antigens across the intestinal epithelium. This may cause a switch from tolerance to immunity, hence representing an increased risk for autoimmune and allergic diseases in individuals whose other genetic determinants, MHC and non-MHC, give rise to inappropriate antigen processing and presentation. In the intestinal epithelium, paracellular permeability is regulated by intercellular TJ proteins. As recently shown, CLDNs are integral TJ components that are critical for maintaining cell-cell adhesion in epithelial monolayers [35-37]. The overall balance of CLDN species expressed in a particular cell type help to define the characteristics of its TJ. For instance, CLDN1 and CLDN4 are postulated to decrease, whereas CLDN2 is postulated to increase TJ-dependent permeability [35]. In line with this notion, and with the appreciation of reduced small intestinal permeability in GS patients, we have shown here that the GS mucosa expresses significantly higher levels of transcripts for *CLDN4 *relative to CD or DC. In contrast, other *CLDN *genes and other genes associated with TJ function measured in this study did not appear to be expressed differently in the GS or CD mucosa compared to controls. Together, these findings suggest that the distinct clinical and serological features between GS and CD patients are associated with marked differences in mucosal barrier function and with apparent differences in the expression of *CLDN4*, which encodes for a critical TJ component [38]. Further studies are required to compare the distribution and assembly of this and other TJ proteins in these conditions.

Patients with GS do not present significant autoimmune or allergic comorbidities, and, as we also have shown here, the serology for common autoantibodies, including anti-tTG IgA, is negative. Interestingly, AGA IgA and IgG were positive in almost 50% of cases. Similarly, higher than expected titers of AGAs, signs and symptoms associated with non-CD gluten sensitivity, have also been reported for schizophrenia [39] and autism spectrum disorders [40]. While in CD there is a strong genetic association with the class II MHC haplotype, with about 95% of patients carrying *HLA-DQ2* and the remaining 5% carrying *HLA-DQ8*, we have shown that only about 50% of patients with GS carry *HLA-DQ2* and/or *HLA-DQ8*, a percentage slightly higher than that in the general population. This suggests a reduced level of involvement of MHC-dependent adaptive immune responses in GS relative to CD. We have further shown that the GS mucosa contains increased numbers of CD3^+ ^IELs, even though these numbers were significantly lower than those in active CD patients in the context of relatively conserved villous architecture, corresponding to the 0 and 1 stages of the Marsh classification. This is in line with a more limited involvement of the adaptive immune system in GS and may explain why this condition is not accompanied by significant autoimmune phenomena.

In CD, an adaptive response has been shown to be triggered by tTG-deamidated gluten peptides bound to DQ2 or DQ8. This involves the mucosal recruitment and activation of Th1 and Th17 clones and the production of Th1- and Th17-associated cytokines, namely, IFN-γ and IL-17A, which contribute to disrupting barrier function and initiating tissue damage [16,18-21,41]. In an earlier report, we showed that IL-17A transcripts are expressed at significantly higher levels in the small intestine mucosa of at least a subgroup of CD patients, but not in GS patients [15]. In this study, we have extended this finding and show that the Th1 signature cytokine, IFN-γ, also is expressed at significantly lower levels in the GS versus CD mucosa. Moreover, in CD but not in GS, we observed a significantly enhanced expression of IL-6, a pleiotropic cytokine that is known to promote the differentiation and function of Th17 cells, as well as a similar trend in the expression of IL-21, consistent with its established role in the pathophysiology of Th1 and Th17 cells.

These findings might indicate that GS is an inflammatory condition mostly supported by innate immune mechanisms. Among these, TLRs represent a family of evolutionarily conserved receptors able to detect microbial invasion via pattern recognition and mediate a rapid inflammatory response which may or may not progress into an antigen-dependent adaptive response. Different combinations of TLRs are expressed in hematopoietic cells and nonhematopoietic cells such as intestinal epithelial cells [42-44]. In this study, we have observed that small intestine expression of TLR2, and to a lesser extent TLR1 but not TLR4, is increased in GS patients. In the absence of markers of adaptive immunity, as we have seen, this suggests a prevalent role of the innate immune system in the pathogenesis of GS.

Taken together, these findings support the idea that the prevalent involvement of innate versus adaptive immune pathways may help explain the clinical and serological differences in GS versus CD patients. Reduced function of Treg cells, and specifically of "adaptive" Treg cells, has been proposed to account for the loss of immune homeostasis and the development of autoimmune responses in CD and related conditions [26]. It might be inferred, then, that Treg could efficiently prevent progression to this response in GS patients. A significantly reduced mucosal expression of the distinctive Treg marker, FOXP3, as appreciated in GS patients in this study, is therefore surprising and counterintuitive in the light of these considerations. However, at least as surprisingly, in several studies FOXP3 and other Treg-expressed molecules, such as TGFB1, have been found to be upregulated in the peripheral blood and intestinal mucosa of patients with CD and related conditions, for example, type 1 diabetes [28,45]. Analogous findings have been reported in other conditions associated with the extensive involvement of adaptive immunity, such as allergy and asthma, leading to speculation that a compensatory expansion and/or mobilization of Treg might take place concomitantly with the buildup of an adaptive effector response [46]. Paradoxically, then, if this assumption is true, a reduced expression of Treg markers in GS might be interpreted in the context of a generally reduced activation of adaptive immunity relative to CD. While more studies obviously are needed to elucidate this issue, a better understanding of Treg function in CD and related conditions will help characterize the possible pathogenetic role of reduced Treg activation and/or recruitment in GS.

## Conclusions

The results of this study suggest that CD and GS are distinct clinical entities caused by different intestinal mucosal responses to gluten. CD results from a complex, and as yet undetermined, interplay of increased intestinal permeability, mucosal damage, environmental factors in addition to gluten, and genetic predisposition, which involves both MHC and non-MHC genes. The typical intestinal lesions in CD are thought to be mediated by both innate and adaptive immune effector pathways. Our findings suggest that, in a different way, GS is associated with prevalent activation of an innate immune response. Although the mechanisms responsible for the loss of intestinal barrier function in CD have been delineated in part, the factors responsible for the loss of gluten tolerance and the development of autoimmunity in this condition are still incompletely understood. We believe that this study could contribute to the clinical characterization of GS as a condition associated with prevalent gluten-induced activation of innate immunity in the absence of detectable changes in mucosal barrier function, and that it provides additional clues to the definition of the complex gluten-induced changes in TJ regulation and immune processes underlying CD pathogenesis. Double-blind, placebo-controlled studies are necessary to further solidify the definition of GS patients and to search for specific biomarkers for a proper diagnosis.

## Abbreviations

AGA: anti-gliadin antibodies; CD: celiac disease; CLDN: claudin; DC: dyspeptic controls; EC: enterocyte; EMA: endomysium antibodies; FOXP3: forkhead box P3; GS: gluten sensitivity; IEL: intraepithelial lymphocytes; IFN: interferon; IL: interleukin; LA: lactulose; MA: mannitol; MHC: major histocompatibility complex; OCLN: occluding; PCR: polymerase chain reaction; TGF: transforming growth factor; Th: T helper; TJ: tight junction; TLR: Toll-like receptor; Treg: regulatory T-cell; tTG: tissue transglutaminase.

## Competing interests

AF is stockholder of ALBA Therapeutics (Baltimore, MD, USA). AS, KML, VC, MC, MTG, MD, RS, GM, CT, AP, MIR, PE, FF, MC, GR and LDM have no competing interests to declare.

## Authors' contributions

AS, GM and AF conceived the study. AF supported the study. AS, KML, VC, MTG, GM, MC, LDM and AF contributed to designing the study. AS and KL carried out most of the molecular genetics studies. MC carried out the molecular assay of Toll-like receptor pathways. MD, CT and GR participated in sequence alignment studies. RS and FF carried out the immunohistochemical assays; MIR and PE participated in the recruitment of the patients and carried out the endoscopy procedures. AS, KML, VC and AF were involved in the analysis and interpretation of the data. AS, KML, VC, GM and AF drafted the manuscript. AS, KML, VC, MD, CT, MC, GR, LDM and AF critically revised the manuscript. All authors gave their final approval of the version of the manuscript to be published.

## Pre-publication history

The pre-publication history for this paper can be accessed here:

http://www.biomedcentral.com/1741-7015/9/23/prepub
